# Neophobia—A Natural Developmental Stage or Feeding Difficulties for Children?

**DOI:** 10.3390/nu14071521

**Published:** 2022-04-06

**Authors:** Agnieszka Białek-Dratwa, Elżbieta Szczepańska, Dorota Szymańska, Mateusz Grajek, Karolina Krupa-Kotara, Oskar Kowalski

**Affiliations:** 1 Department of Human Nutrition, Department of Dietetics, Faculty of Health Sciences in Bytom, Medical University of Silesia in Katowice, ul. Jordana 19, 41-808 Zabrze, Poland; eszczepanska@sum.edu.pl (E.S.); okowalski@sum.edu.pl (O.K.); 2 Poradnia Żywienia Dzieci w Bielsku Białej/Child Nutrition Clinic in Bielsko-Biała, 43-309 Bielsko Biała, Poland; poradniazywieniadzieci@gmail.com; 3 Department of Public Health, Department of Public Health Policy, Faculty of Health Sciences in Bytom, Medical University of Silesia in Katowice, ul.Piekarska 18, 41-902 Bytom, Poland; mgrajek@sum.edu.pl; 4 Department of Epidemiology, Faculty of Health Sciences in Bytom, Medical University of Silesia in Katowice, ul. Piekarska 18, 41-902 Bytom, Poland; kkrupa@sum.edu.pl

**Keywords:** feeding neophobia, feeding difficulties, dietary expansion, child nutrition, food selectivity

## Abstract

Food neophobia is the tendency to reject or be reluctant to try new and unfamiliar foods. Due to the period of its occurrence, which falls in the years of early childhood, it can significantly affect the child’s food choices, shape taste preferences, and significantly influence the quality of the child’s diet. The neophobic attitude has an important evolutionary significance because it protects the individual from ingesting potentially dangerous substances. On the other hand, it fosters avoidance behaviors that can also relate to the beneficial aspects of obtaining and consuming food. Currently, the strong emphasis placed on food safety means that neophobia may be less adaptive; nevertheless, a conservative attitude toward new foods still prevails. There is a strong association between food neophobia and the diversity of a person’s diet and previous exposure to different foods. This review describes behaviors associated with food neophobia and analyzes other feeding and eating difficulties in children that should be differentiated from food neophobia. Management approaches affecting the reduction in food neophobia in children through various dietary and psychological interventions are also proposed.

## 1. Introduction

An increasing number of parents come to dieticians with problems concerning the feeding and nutrition of their children. Failure in expanding the diet or a sudden (in the parents’ opinion) discontinuation of eating various dishes makes them increasingly worried about their child’s diet and eating behavior. Eating behavior is very individual and depends on many factors. In the case of young children, the readiness of the child to accept solid foods acquired oral–motor skills, taste preferences, etc., significantly affect feeding behavior. In the case of parents, it is important to understand that children are at the learning stage and are only just developing their taste preferences and self-regulatory processes and acquiring the skills of solid food intake [1,2,3].

Feeding seems to be a simple activity. However, it is a complex process that requires the interaction of the central nervous system with the peripheral nervous system, a properly functioning oropharyngeal mechanism, an efficient respiratory system, and a properly functioning digestive system. During feeding, coordination of the muscles and structures in the oral cavity with the cranial and spinal nerves is essential. This is necessary for proper coordination of the lips, jaw, cheeks, tongue, and soft palate. To prevent aspiration of ingested food into the airways, it is necessary to coordinate the functions of sucking—and in older children, taking in solid food, breathing, and swallowing. All of these complicated processes in young children, who are still being fed by their parents or caregivers, additionally require properly coordinated interactions between them. Otherwise, feeding problems may arise [4]. These problems are biopsychosocial [5], as both physiological and psychosocial factors contribute to their initiation and maintenance. The causes of feeding difficulties may also include motor and sensory disorders in the orofacial sphere such as oral sensory–motor disorders [6,7,8], large and small motor disorders, motivation (congenital or acquired), which may result in poor growth or weight gain [9,10,11,12], as well as individual predispositions that will affect the willingness to try new tastes and textures of food [1].

The aim of this study was to analyze the nutritional problem of neophobia among children. To identify the risks of nutritional neophobia in infancy and childhood, and to identify possible dietary and psychodietary interventions to expand the diet of young children with nutritional neophobia.

We reviewed electronic databases including PubMed, ScienceDirect, Web of Science, and Google Scholar, over the last 10 years (2012–2022). However, due to the lack of complete data and the tools used to assess nutritional neophobia, we extended the literature review to 1990.

The following inclusion criteria were used for the review: articles in English and in Polish. The following keywords were used to search for articles: neophobia (623 articles from 2012 to 2022 and 949 articles from 1990 to 2022), child feeding difficulties (2030 articles from 2012 to 2022 and 3258 articles from 1990 to 2022), and child food selectivity (5970 articles from 2012 to 2022 and 9665 articles from 1990 to 2022).

The literature review included comparative studies, cross-sectional studies, and randomized controlled trials. The last search was run on 10 January 2022.

## 2. Epidemiology of Food Neophobia

**Figure 1:**
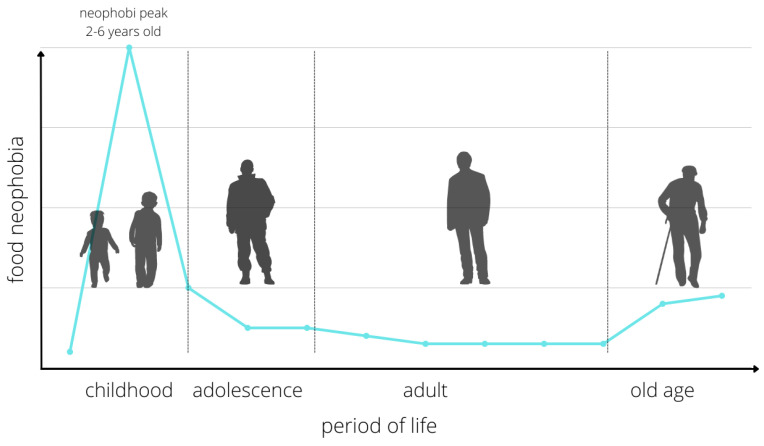
Changes in the level of nutritional neophobia in different periods of life. Source: Own elaboration based on Kozioł-Kozakowska and Piórecka (2013, p. 2).

Difficulties in defining and differentiating food neophobia pose problems in obtaining epidemiological data. According to Faith et al., it affects 40% of children aged 4–7 years diagnosed with CFNS [13]. In a study by Antoniou et al., the problem affected 14% of children aged 5–9 years (CFQ) [14]. In contrast, Johnson et al. diagnosed neophobia in 44% of 4-year-olds (CFNS) [15]. The most sensitive period for neophobic behavior toward food is observed in children before the age of 2 years. However, the emergence of neophobia earlier is not excluded—even in the first year of life the so-called early neophobia. Between the ages of 2 and 6, when an increase in the child’s independence and autonomy concerning food choices is observed, one speaks of so-called developmental neophobia, which is a natural stage of development (Figure 1).

In a Polish population-based study, one in every tenth child was diagnosed with high levels of food neophobia, with the highest percentage observed in the age group of 5–6 years. Most studies confirm that the occurrence of food neophobia is independent of gender [16,17]. To compare the relative contribution of genetic and environmental influences on the occurrence of neophobia, studies of pairs of pediatric twins were conducted. The results revealed a significant contribution of genetic factors to the development of food neophobia, with heritability estimated at 78% in children aged 4–7 years and 72% in children aged 8–11 years. The only study on adult twins showed a comparably high heritability [18].

## 3. Food Neophobia

The terminology of feeding, eating, and nutritional problems is relatively extensive. Food selectivity, food neophobia, picky eating, food aversion, and food avoidance/restriction are all terms that have one common denominator for parents—“my child does not eat”. However, there is much more to this term than the mere fact of not eating enough (in the parents’ opinion), which is very often heard by dieticians in their offices [3,12].

The terms that describe problems related to food intake are not the same. Some of these terms include eating and nutrition disorders, some include difficulties with food intake of various etiologies, and some refer to behaviors that are a natural developmental stage in the child’s life. Incorrectly interpreted—both by parents and specialists—they introduce chaotic behavior, which, inadequate to the condition, may cause serious health consequences or reinforce undesirable behaviors [19].

Eating behavior is determined by many factors, including biological, anthropological, economic, psychological, socio-cultural, and home-related factors, and their influences compete, reinforce, and interact with each other and are shaped by the individual situation of the family [20].

One of the child’s eating behaviors that cause parents great concern is food neophobia. Food neophobia is characterized by the child’s rejection of foods that are new or unfamiliar, both visually and in terms of taste [21,22]. Neophobic behaviors can appear, to a small extent, as early as the first year of life, but most often intensify between 18 and 24 months of age, which is related to the child’s increased mobility. This is a stage that should eventually resolve spontaneously. It is worth noting that the period of food neophobia overlaps, as it were, with the time when the child’s rate of growth and development begins to slow down. It is also the period when the child begins to express its autonomy, often during a meal. The severity of food neophobia changes throughout the individual’s life and is modulated by various factors. It manifests itself most in children and probably prevents them from experimenting and thus experiencing different foods [3,7,11].

According to the Chatoor classification, food neophobia is included in the group of feeding disorders—selective eating. Selective eating, in turn, is included in the broader group that is sensory food aversions [23].

Feeding disorders occur when a child refuses or avoids eating and is unable to eat due to behavioral disorders, neurological disorders, anatomical abnormalities of the gastrointestinal tract, as well as comorbidities of the cardiovascular system, respiratory system, genetic, metabolic, or allergic diseases [4]. Feeding disorders consequently lead to health deterioration as a result of chronic insufficiency of nutrients and energy necessary for development. Feeding disorders may require pharmacological treatment and often parallel therapy, carried out by a multidisciplinary team that should include a pediatrician/gastroenterologist, a clinical dietician, a psychodietitian, a neurologist, and a psychologist.

Food neophobia, which is a natural stage of development, requires neither treatment nor therapy if it runs its natural course. However, it requires education from a specialist and understanding on the part of the parent. Then, without reinforcing the undesirable parental behaviors, this stage spontaneously passes [3,7,22].

To some extent, the conceptualization of feeding disorders, feeding difficulties, and feeding behavior problems in children up to 3 years of age was tackled by Kerzner et al. [22]. This new insight allows both professionals and parents to distinguish between what requires intervention in the form of treatment or therapy and what is still the developmental norm [22] (Table 1 and Table 2).

A practical approach to identifying feeding difficulties by taking a broad view of aspects related to organic and behavioral factors makes it easier for professionals working in this area to correctly identify and classify the feeding problems with which concerned parents come to the practice [22].

## 4. Causes of Food Neophobia

The source of food neophobia can be traced back to evolution when a neophobic attitude protected mammals from consuming potentially poisonous food [24]. As an omnivorous species, to survive, humans had to distinguish between safe and poisonous food. Although this ability has lost its value today, it can still be observed in children around 2 years of age (sometimes earlier), when unfamiliar foods or foods served differently than before cause anxiety in the child, and a relative preference for familiar foods is apparent [25,26].

Although neophobia is genetically determined, it is above all environmental factors that underlie individual differences in taste preferences [25,26]. The genetic factors influencing food choice involve taste receptors, which can influence the differential perception of sweet, umami, or bitter tastes, depending on differences in individual genes. Hence, some children are more tolerant of bitter-tasting green vegetables such as broccoli or cabbage, while others will not be bothered by them, and some will reject these foods at the mere sight of them [26].

Food neophobia can be inherited or shaped by the environment in which the child is raised. The social environment, family, and peers, may allow children to show their personality through eating behavior and thus follow their tendency toward food neophilia or neophobia [27]. Parents, predominantly mothers, who play a crucial role in shaping children’s eating behavior [28], may pass on the tendency toward food neophobia to their children [29]. Therefore, understanding food neophobia in adults is important to prevent its health effects in this group but also because of its possible impact on people, especially their children [30].

Food preferences are highly variable, with the result that aversion to eating new foods and those less accepted may be reduced in the child. This is influenced by several different factors, which include the diet during pregnancy and lactation [31], or the mode of exposure and its repetition [26]. These are important factors that may indirectly influence feeding difficulties and the course of food neophobia, which, depending on individual characteristics, may proceed unnoticed [26,31].

Sensory characteristics have been singled out as one of the most influential determinants of eating behavior, and among these, textures are the main reason for food rejection or acceptance in children [32,33,34]. Certain textural characteristics have been shown to induce food rejection through disgust even before tasting in children [25]. The sounds accompanying the disintegration of food in the oral cavity, due to their texture and structure, can influence its acceptance or lack thereof [35]. Regarding foods, hard, lumpy, and grainy textures are generally less acceptable to children of all ages [34]. Sensory sensitivity, also known as sensory over-reactivity, can be defined according to an individual’s response to sensory information, including, but not limited to, taste, touch, sight, and smell. This phenomenon can be considered part of the pickiness that causes children to accept less variety in foods presented by their parents and loved ones [35].

Food preferences and aversions are shaped by the chemosensory system that underlies taste perception [36]. The mechanism through which we learn and modulate food preferences and aversions is represented by repeated exposure, but only if it involves actual tasting [37]. Food neophobia appears to be an extremely complex attitude, the strength of which changes throughout life and is modulated by many different factors [38]. The quality of a person’s diet is strongly influenced by their attitudes toward food (and new foods in particular) and has a considerable impact on their health and well-being [39].

One of the most important factors in the whole process of feeding is the feeding style used. As shown in a study by Meijing et al. [40], the following factors mainly influence the course of neophobia: urging the child to eat with a definite refusal from its side, unpleasant emotions during the meal (e.g., nervousness of the parent, stress, crying of the child), and high level of neophobia in the mother. Similar conclusions were reached by de Oliveira Torres et al. In a systematic review of the literature, stating that the level of neophobia in children is influenced by the eating habits of the parents, the inborn preference of children for sweet and salty tastes, the inadequate consistency to the child’s psychomotor skills, parental pressure during meals, the failure to read signals concerning hunger and satiety or monotony in child nutrition, etc. [41].

Food neophobia, on the one hand, is a natural stage in development, while on the other, its occurrence can influence the perpetuation of inappropriate behaviors. Therefore, if the neophobic behavior does not disappear but even becomes more intense, appropriate intervention should be undertaken. Factors influencing food neophobia are very diverse; therefore, as in the case of eating disorders, the patient should be dealt with by a team of specialists including a pediatrician/gastroenterologist, clinical dietician, neurologist, psychologist, sensory integration therapist, feeding therapist, etc. [34,35,40,41].

## 5. Differential Diagnosis of Feeding Difficulties

In the process of diagnosis related to feeding difficulties, the use of the Montreal Children’s Hospital Feeding Scale (MCH-FS) may be helpful [42]. The MCH scale related to child feeding is the first scale of its kind to be used for screening children with feeding challenges, for both prevention and diagnosis. It can be used as a very good tool to identify possible feeding difficulties in children from 6 months to 6 years of age. The MCH scale consists of 14 questions addressing issues related to the meal pattern, appetite assessment, meal duration, problems within the orofacial sphere, or the parent’s perception of the child’s correct weight and height [42].

The following questions were used in the Polish adaptation: How do you assess your child’s meal pattern? How worried are you about your child’s meal pattern? How do you assess the appetite (feeling of hunger) of your child? At what point during a meal does your child start refusing to eat? How long do your child’s meals last (in minutes)? How do you assess your child’s behavior during meals? Does your child choke, choke, spit up or vomit at certain types of food? Does your child keep food in the mouth without swallowing it? Do you have to walk behind your child or distract him/her (toys, TV) to get him/her to eat? Do you have to force your child to eat or drink? How do you evaluate your child’s chewing (or sucking) skills? How do you rate your child’s growth (weight, height)? How does feeding your child affect your relationship with your child? How does feeding your child affect your family relationships? [42].

Each question is answered on a 7-point Likert scale [1]. The score obtained after an appropriate conversion of the points (there is no Polish version) allows the interpretation of difficulties on three levels: mild difficulties, moderate difficulties, and severe difficulties [1]. The Polish adaptation of the MCH-FS scale seems to be a suitable tool for the assessment of feeding difficulties such as feeding neophobia [1,42].

In practice, the Food Neophobia Scale for Children (FNSC) [43] is used to determine the level of food neophobia in young children, or the Food Situations Questionnaire (FSQ) [44] is used to self-assess food neophobia in children from 7 to 12 years of age, in which the differential diagnosis concerns must-eat disorders. However, it has not been translated into Polish and adapted to the diet in Poland. The FNSC consists of 10 statements on neophobic behavior, with respondents agreeing or disagreeing. The interpretation of this scale is difficult, due to the lack of an update on the so-called “new foods” that are currently available but were not common when the FNSC was developed. It is possible to consider whether the FNSC can capture what is currently considered novel foods, especially referring to food exports and imports. Additionally relevant is the meaning of which “ethnic” foods are no longer considered novel, e.g., pizza, sushi, the tortilla, all of which have become part of the daily diet [44,45].

## 6. Impact of Neophobia on Child Health

In the past, neophobic behavior toward food served to make humans, as omnivorous species, feel safe in an environment full of plants with toxic properties and dangerous bacteria that could reproduce in spoiled food. Currently, in the modern world, neophobia is a mechanism needed in the period of early childhood; when the child learns about the world also through the sense of taste, the attitude of distrust toward novelty protects the child from the danger of eating something potentially dangerous to health. As the child grows, biological instincts are masked by learned behaviors, so the neophobic attitude toward food begins to have negative effects, causing a reduction in the consumption of a variety of products, which may translate into diet quality [17].

Nutritional neophobia can lead to deficiencies of certain essential nutrients, especially vitamins and minerals. This is evidenced by numerous studies that report, among other things, that the diet of children with high levels of neophobia is not very varied. Neophobic children eat less than the recommended levels of vegetables, fruit, and dairy products. In addition, children who eat only selected products may not acquire some of the skills associated with eating promptly, especially if they only eat foods with a soft texture or consistency of mush [43,46,47,48,49].

The negative health consequences of nutritional neophobia should be considered in terms of lost potential health benefits resulting from a poor, poorly varied diet and, above all, eating too few vegetables and fruits, compared with the recommendations. Neophobia of nutrition also gains importance in the context of the popularized concept of nutritional programming, understood as a long-term or life-long effect of a stimulus or signal affecting structures or functions of the organism during a critical period of development. The occurrence of factors such as malnutrition, deficiency, or excess of certain nutrients during so-called critical periods can reprogram the metabolism, thus leading to irreversible health consequences [3,25].

Neophobia is a considerable problem in the case of children with a food allergy or intolerance, overweight or obesity, and those who suffer from diseases in which it is necessary to follow a special diet. These children are forced to follow a diet, and their unwillingness to try new products makes it difficult to implement dietary recommendations, which can have an impact on the course of the disease. Particularly noteworthy is the fact that proper nutrition is not able to completely compensate for the previously lost opportunities for optimal physical and mental development until later in life [17].

## 7. Management of Children with Nutritional Neophobia

Whatever the reason, a child with intake difficulties should receive dietary care. It should take into account the dynamics of the nutritional status, the current nutritional value of the diet, and the nutritional and medical history. The MCH scale can be used for the interview, and the interview can be expanded with self-made questions resulting from the initially described problem [1,42].

In the case of food neophobia in the child, education delivered in an empathetic and guilt-free manner to the parent may be sufficient. Parental behavior is very often driven by a concern for the child’s health, and this should be borne in mind when guiding the parent. If an eating disorder or feeding difficulties are suspected to be due to organic causes, consultation with individual specialists is necessary [3,17,50,51].

The management of children with feeding neophobia should include several different factors: meals should be given in small amounts and at fixed times, the food product/food should be offered to the child repeatedly but without applying pressure, attempts should be made to leave the food within the child’s reach without necessarily offering it to them, and the new product should be given in the company of familiar products using the food chaining method [3,50,51]. Food chaining is a method helpful in feeding therapy for patients with eating disorders. Initially, the diet should be based on products known to the child but in a different way than before (e.g., changing the shape of pasta) or changing the form of administration (e.g., cutting fruit instead of blending). These products are treated as already known to the patient, but it is necessary to gradually change the routine and start introducing various forms of their administration [50,51].

During taming therapy, available gadgets can be used, including animal-shaped spoons or forks, bowls, plates with well-known fairy tale characters, and books on the theme of food. It is also necessary to ensure a positive atmosphere during the meal (e.g., not complaining because of eating too little, not comparing to other children, not using rewards in the form of sweets for eating new products, etc.). Additionally, the possibility of independent eating, in young children also with hands, should be given using the baby-led weaning (BLW) method. The main idea of the BLW method is to give solid food to the child and give them the possibility of choosing and eating it on their own [52]. Trying a new product may be rewarded with praise or playing together but not with sweets. In situations of frequent choking, it is worth consulting a neurologist [17,52].

It is also worthwhile to allow children to participate in the preparation of meals, as this influences, among other things, their preference for products, reduces their sense of anxiety, and builds positive relationships with their caregivers. In addition, children who help with cooking at home more often show a greater preference for eating fruit and vegetables [2,53,54,55].

The social environment is a recognized determinant of children’s behavior [56]. Parents, siblings, and teachers are also important members of this environment and together influence the development of their eating behavior. Therefore, it should be borne in mind that eating behavior should also be correct in the environment in which a neophobic child resides, i.e., based on the consumption of all food groups but without exerting pressure on the child [2,57,58].

Developmental neophobia is a stage in a child’s life that should pass spontaneously. Occasionally, however, this behavior is so strong and fixed that it can accompany the child also in their school years until adulthood. It mostly affects children with various disorders, so it is difficult to speak of developmental neophobia. Such an attitude suggests food selectivity, food aversion, or an eating disorder, e.g., avoidant restrictive food intake disorder (ARFID) in a child; therefore, specialist intervention is necessary in such a case. The reason for a restricted diet may not be in the older child’s fear of the new but due to coexisting illnesses, disorders, or other causes that should be diagnosed as soon as possible.

## 8. Conclusions

It would seem that food neophobia is a well-studied problem and, in some cases, should be treated as a completely physiological phenomenon. However, it is worth noting here that several studies conducted focusing on the causes of neophobia emphasize the psychological context of this behavior. Food avoidance or food selectivity in childhood can lead to many consequences in the future, related to both physical and mental health. Psychodietetic management of food neophobia consists mainly of familiarization with new foods, their appearance, taste, texture, and the systematic introduction of newer and newer variants of food. It also involves working with parents and caregivers to explain the phenomenon of neophobia, as a better understanding of this behavior allows for a milder course of the disease. The methods described in this study do not exhaust the range of therapeutic possibilities but constitute a collection of the most effective and best-discussed methods in the professional literature. Neophobia is a natural developmental stage in children aged 2–6 years; however, if the neophobic behavior does not disappear but actually increases, an appropriate intervention should be undertaken in order not to lead to more serious consequences related to the restriction of eating a variety of foods. The patient should then be managed by a team of specialists including a pediatrician/gastroenterologist, clinical nutritionist, neurologist, psychologist, sensory integration therapist, and nutritional therapist.

## Figures and Tables

**Table 1:** Classification of feeding difficulties according to Kerzner et al. in terms of limited appetite and food selectivity (own elaboration) [22].

Limited Appetite	Food Selectivity
A misperceived problem: resulting from excessive parental concern despite the child’s normal development; The perception of a child with a smaller height as an “underachiever”; The use of inappropriate feeding practices (e.g., pressure, force feeding).	A misperceived problem: Food neophobia: perceived by parents as selective eating, with the idea that children eat too little variety of foods. Neophobia is a natural stage of development, reaching its peak between 18 and 24 months. New foods begin to be accepted after repeated exposure.
Temperament: An energetic child: a type of child who is active, energetic, and curious about the world. With the transition to independent eating the child “does not have time” to eat. They do not want to sit during the meal and eat less food, which contributes to slower growth. No organic cause. Characteristic is the conflict between parents and child, which unresolved limits the child’s cognitive potential. Apathetic, withdrawn child: children are inactive, uninterested in eating, appear unresponsive, and without proper communication with the parent. Both child and caregiver are depressed, and interaction is lacking. There is a high risk of malnutrition, anorexia, and depression in these children.	Mild food selectivity: includes a group of children classified as “picky eaters”. Despite trying different foods, their dietary repertoire is poorer than that of other children; These children develop normally, while “picky” food, as perceived by the parents, can cause conflicts related to food forcing and inappropriate behavior. Very high food selectivity: There is a real risk of nutritional deficiencies; A diet limited to 10–15 foods; Selectivity may be due to food aversions; Selectivity may be due to disorders of sensory modulation; Characteristic of children with autism.
Organic causes gastroenterological disorders (eosinophilic esophagitis, reflux, constipation, gastritis); Cardiopulmonary disorders; Neurological disorders; Metabolic disorders; Structural anomalies.	Organic causes: Developmental delays; Dysphagia.

**Table 2:** Classification of feeding difficulties according to Kerzner et al. in terms of fear of food/feeding (own elaboration) [22].

Fear of Food/Feeding
Children with fear of feeding are usually those with food aversions or who have experienced unpleasant (e.g., choking) or painful (e.g., tube feeding) feeding situations. Anxiety may also accompany the first attempts to feed solids and is caused by inexperience, undeveloped psychomotor skills, and an unfamiliar situation.
A misperceived problem: Infant crying is perceived as a sign of hunger or fear at the sight of the breast or bottle; However, crying can be the result of high sensitivity in these babies or colic; These children usually take adequate amounts of food.
Fear of feeding in infants: These babies initially eat willingly, but after a short time, they become restless, cry, and move away from the bottle or the breast; These infants do not show such symptoms during night feeding; Over time, at feeding time, they begin to present a jarring fear at the very sight of the breast, bottle, or feeding chair.
Fear of feeding in older children: Visible in children who choke while eating, or who vomit during meals; There is a refusal to take solid food; Can be a consequence of pressure and force feeding by parents; Referred to as functional dysphagia, or phagophobia.
Organic causes: Odynophagia; Gastroparesis; Visceral hypersensitivity; Feeding by nasogastric tube.
Feeding styles: Responsive; Controller; Permissive; Negligent. The last three styles are related to the risk of inappropriate behavior among parents. Inappropriate parental feeding behavior can have an impact on feeding difficulties, thus indirectly influencing feeding disorders. The most desirable feeding style is the responsive style.

## Data Availability

Not applicable.
